# Multivariate associations between sleep patterns and self-regulation in adolescence: Canonical correlation analysis across ABCD and NCANDA cohorts

**DOI:** 10.1080/07420528.2026.2685032

**Published:** 2026-06-30

**Authors:** Tommy Gunawan, Ty Brumback, Brant P. Hasler, Qingyu Zhao, Susan F. Tapert, Alejandro D. Meruelo

**Affiliations:** a Department of Medical and Clinical Psychology, Uniformed Services University of the Health Sciences, Bethesda, Maryland, USA; b Henry M. Jackson Foundation for the Advancement of Military Medicine, Bethesda, Maryland, USA; c School of Psychology, Xavier University, Cincinnati, Ohio, USA; d Department of Psychiatry, University of Pittsburgh, Pittsburgh, Pennsylvania, USA; e Department of Radiology, Weill Cornell Medicine, New York, USA; f Department of Psychiatry, University of California, San Diego, La Jolla, California, USA

**Keywords:** Adolescence, sleep, chronotype, self-regulation, circadian misalignment, impulsivity, ABCD, NCANDA

## Abstract

Adolescence involves pronounced changes in sleep timing and duration that have been linked to executive functioning, impulsivity, and risk behaviors, yet evidence for generalizable sleep – selfregulation associations remains limited. We used regularized canonical correlation analysis to examine multivariate relationships between sleep and self-regulation across two independent cohorts. In the Adolescent Brain Cognitive Development study (*n* = 11,143), later chronotype and shorter sleep duration were associated with higher impulsivity, particularly urgency and deficits in planning and perseverance (*r* = 0.21, permutation *p* < 0.001), with minimal contribution from delay discounting. Social jetlag showed weak loadings. In the National Consortium on Alcohol and Neurodevelopment in Adolescence cohort (*n* = 831), only one canonical function was statistically significant (*r* = 0.15, *p* = 0.011), characterized by later chronotype and greater social jetlag, with minimal contribution from sleep duration and primary loadings on impulsivity-related measures. Applying the ABCD-derived sleep – self-regulation pattern to NCANDA produced a small but nonzero association, driven primarily by social jetlag and delayed sleep timing, with minimal influence of sleep duration. These findings provide multivariate evidence linking delayed sleep timing and circadian misalignment to adolescent self-regulation, though effect sizes attenuate across cohorts. Longitudinal work is needed to clarify predictive pathways and inform sleep- focused interventions during this vulnerable developmental period.

## Multivariate Associations Between Sleep Patterns and Self-Regulation in Adolescence (Graphical Abstract)

Created in BioRender. Meruelo, D. (2026) https://BioRender.com

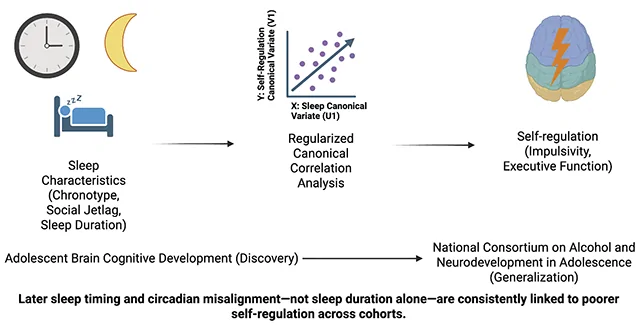

## Introduction

Adolescence is a developmental period marked by profound neurobiological, cognitive, and behavioral changes (Giedd et al. 1999; Meruelo et al. 2017). Among the factors shaping this trajectory, sleep plays a central role (Anastasiades et al. 2022). Circadian timing, sleep duration, and sleep regularity are increasingly recognized as critical determinants of cognitive development, emotional regulation, and health outcomes during this vulnerable window (Crowley et al. 2007; Hagenauer et al. 2009; Logan et al. 2018). Across development, normative changes in circadian biology include a progressive delay in the timing of the endogenous circadian system from late childhood through adolescence, accompanied by later sleep – wake preferences (“eveningness”), reduced sleep duration on school nights, and increased night-to-night variability in sleep timing (Roenneberg et al. 2004; Gradisar et al. 2011; Crowley et al. 2018). These shifts are thought to reflect both biological changes in circadian phase and sleep homeostasis and increasing psychosocial demands, culminating in a “perfect storm” of sleep vulnerability during adolescence (Crowley et al. 2018, 20).

A growing literature indicates that later chronotype (a tendency toward later sleep – wake timing, often referred to as eveningness) and greater sleep irregularity are associated with poorer executive functioning, heightened impulsivity, and increased risk-taking in youth. Social jetlag – the misalignment between endogenous circadian timing and externally imposed schedules, typically operationalized as the difference in midsleep timing between school/workdays and free days – has been repeatedly implicated in these associations. Adolescents are particularly susceptible to social jetlag due to the normative developmental delay in circadian timing that emerges after puberty and peaks in late adolescence or early adulthood, combined with early school start times during middle school and high school (Roenneberg et al. 2004; Gradisar et al. 2011; Kennaway 2023). Evidence from the Adolescent Brain Cognitive Development (ABCD) Study (Volkow et al. 2018) and the National Consortium on Alcohol and Neurodevelopment in Adolescence (NCANDA) study (Brown et al. 2015) and other large adolescent samples demonstrates that eveningness and social jetlag are linked to deficits in inhibitory control, and some studies report associations with steeper delay discounting and more impulsive decision-making (Hasler et al. 2022; McCarthy et al. 2023; Gelino et al. 2025).

Despite these advances, most prior investigations in adolescent populations have evaluated individual sleep features in relation to individual outcomes using regression-based approaches. Such designs may obscure broader patterns of covariation – that is, how constellations of sleep dimensions jointly relate to cognitive and behavioral regulation. This limitation is notable given growing recognition of sleep as a multidimensional construct, encompassing timing, regularity, duration, and subjective quality, rather than a single attribute (Buysse 2014; Meltzer et al. 2021). In adult samples, multidimensional approaches – including machine-learning and multivariate methods – have demonstrated that combinations of sleep characteristics provide stronger and more informative associations with health outcomes than any single sleep dimension alone (Wallace et al. 2019). Comparable multivariate approaches remain less common in adolescent sleep research.

These hypotheses are consistent with developmental and neurobiological models of adolescent selfregulation. The dual-systems model posits a relative imbalance between earlier-maturing reward-related processes and later-developing cognitive control systems, increasing vulnerability to impulsive behavior during adolescence (Casey et al. 2008; Steinberg 2008; Somerville and Casey 2010). Complementary models of sleep and emotion regulation suggest that sleep disruption and circadian misalignment impair prefrontal control and heighten affective reactivity (Yoo et al. 2007; Walker 2009; Goldstein and Walker 2014), thereby biasing behavior toward urgency-driven impulsivity. These frameworks collectively predict that multivariate patterns of sleep disturbance will be more strongly linked to impulsivity than to other forms of decision-making, a prediction we evaluate using canonical correlation analysis.

Here, we use the term self-regulation to refer to capacities including inhibitory control and working memory (cognitive regulation), as well as impulsivity and related behavioral tendencies (behavioral regulation). Studying these outcomes during adolescence is particularly important: this developmental period involves rapid maturation of frontostriatal circuits, increasing autonomy and exposure to irregular schedules, and heightened vulnerability to dysregulated behavior. Dysregulation in these domains prospectively predicts academic difficulties, injuries, initiation and escalation of substance use, and common mentalhealth problems (Steinberg 2008; Squeglia et al. 2009; King et al. 2011; Moffitt et al. 2011; McLaughlin et al. 2012; Chassin et al. 2016). Yet replication across independent cohorts remains limited, leaving questions about generalizability and reliability unresolved.

Canonical correlation analysis (CCA) (Hardoon et al. 2004; Witten et al. 2009; Nakua et al. 2024) provides a powerful multivariate method to address this gap by identifying latent dimensions of shared variance between sets of sleep and self-regulation measures. CCA can reveal multivariate patterns that are not apparent when examining individual associations, thereby advancing understanding of how multiple facets of sleep collectively map onto self-regulatory capacities. Although data-driven and multivariate approaches are increasingly being applied to adolescent sleep within large cohorts such as ABCD (McCurry et al. 2024), CCA itself has seldom been used to examine sleep – selfregulation relationships in adolescence, and existing applications have largely been confined to single datasets without independent replication.

The present study leverages two large, complementary cohorts to test multivariate relations between sleep and self-regulation and to evaluate their generalizability. We designate ABCD, a nationally distributed sample of over 11 000 adolescents with harmonized measures and comparatively low missingness, as the discovery cohort to maximize statistical power, stability of regularization, and precision of effect estimation. We then evaluate generalizability in NCANDA (Brown et al. 2015), an independent cohort with distinct ascertainment and demographics. Importantly, the cohorts are harmonized at the construct level rather than by identical instruments, with projection analyses restricted to directly overlapping sleep and impul- sivity measures across cohorts: ABCD includes Munich Chronotype Questionnaire (MCTQ)-based chronotype, social jetlag, and sleep duration, whereas NCANDA derives parallel indices of sleep timing/circadian preference, social jetlag, and sleep duration from the Sleep Habits Questionnaire, alongside overlapping selfregulation measures. We first apply regularized CCA in ABCD and then project the resulting ABCD loadings into NCANDA to test cross-cohort generalizability under differences in measurement. We hypothesized that a canonical dimension would link later sleep timing and shorter sleep duration to poorer self-regulation, particularly higher impulsivity and aspects of executive function, and that these associations would be robust to adjustment for age, sex, socioeconomic status, and site.

## Methods

### Participants

Data were drawn from two large-scale, longitudinal studies of adolescent development: the Adolescent Brain Cognitive Development (ABCD) Study (Volkow et al. 2018) and the National Consortium on Alcohol and Neurodevelopment in Adolescence (NCANDA) (Brown et al. 2015). The ABCD Study recruited 11 878 youth ages 9–10 from 21 sites across the United States and assessed them at baseline with extensive behavioral, neurocognitive, and environmental measures. NCANDA is a multisite longitudinal cohort initiated to examine trajectories of neurodevelopment in relation to alcohol exposure and includes 831 participants who were recruited beginning at ages 12–21. Table 1 summarizes baseline sociodemographic characteristics for the baseline analytic cohorts used in the present study, which comprised 11 143 ABCD participants after restricting to the baseline visit and selecting one baseline record per participant, and 831 NCANDA participants. The difference between ABCD baseline enrollment and the analytic cohort reflects harmonization and baseline visit eligibility for the variables used in the present analyses, rather than substantive exclusions.

For the present analyses, we focused on baseline sleep and self-regulation measures. At the level of observed data, 8,567 of 11, 143 (76.9%) ABCD participants and 284 of 831 (34.2%) NCANDA participants had complete data on all core sleep and self-regulation variables. To avoid bias associated with complete-case restriction and to retain maximal power, our canonical correlation analyses were conducted on the full baseline analytic cohorts in each study. Missing data were addressed using cohort-specific imputation strategies. In ABCD, missing values were imputed using multivariate imputation by chained equations with the mice package (van Buuren and Groothuis-Oudshoorn 2011). In NCANDA, missing values were handled using within- visit median imputation prior to model fitting. This approach balanced computational tractability and data characteristics across cohorts, given the larger sample size and broader missingness patterns in ABCD and the smaller sample with higher missingness in NCANDA. Harmonization procedures ensured comparable variable definitions across the two cohorts (Supplementary Table 1).

To further characterize missingness, we examined variable-level missingness rates and their associations with key covariates (Supplementary Table 2). In ABCD, missingness was variable-specific, with higher rates observed for certain NIH Toolbox tasks and more moderate levels for sleep and impulsivity measures. Missingness was systematically associated with observed covariates, particularly age and study site, consistent with a missing-at-random framework and supporting the use of MICE. In NCANDA, missingness was concentrated primarily in the sleep variables, whereas selfregulation measures exhibited minimal missingness; missingness in sleep variables was associated with socioeconomic status but not with age or sex, again consistent with missing-at-random assumptions. Multicollinearity was evaluated using variance inflation factors (VIF), which were within acceptable ranges across both datasets, indicating no evidence of problematic collinearity (Craney and Surles 2002).

### Data Harmonization

Data preparation focused on creating construct-level comparability rather than forcing one-to-one variable matches. For each cohort, standardized preprocessing pipelines were implemented to clean, residualize, and structure data, and to ensure that measures feeding into the CCA reflected parallel domains even when instruments differed.

For ABCD, a harmonization script compiled raw files from the NIH Toolbox (Weintraub et al. 2014; Holdnack et al. 2017), MCTQ (Erdoğan et al. 2022; Wang et al. 2023), UPPS-P (Cyders et al. 2014), and delay discounting (Odum 2011), along with demographic files capturing age, sex, socioeconomic status, and site. Variables were cleaned and restricted to baseline. For NCANDA, an analogous script assembled neurocognitive performance measures (Stroop, Paced Auditory Serial Addition Test or PASAT) (Gronwall 1977; Smolker et al. 2022), Behavior Rating Inventory for Executive Function (BRIEF) subscales (Gioia et al. 2000; Huizinga et al. 2023), UPPS-P, self-reported sleep measures (Sleep Habits Questionnaire), and demo- graphic/site information.

Because ABCD and NCANDA use different instruments in several domains, harmonization operated at the level of domains/constructs (e.g. executive function, sleep disturbance, impulsivity, and related decisionmaking constructs) rather than matching specific variables. Only constructs with strong item-level equivalence (e.g. UPPS-P subscales) were directly aligned. Domains lacking direct counterparts – for example, MCTQ-derived midsleep timing in ABCD or RCFT measures unique to NCANDA – were retained only within their respective cohort analyses. A detailed side- by-side mapping of all ABCD and NCANDA measures, including unmatched domains, is provided in Supplementary Table 1.

### Measures

#### Sleep Variables

In ABCD, chronotype (sleep corrected), relative social jetlag (sleep corrected; SJLsc), and sleep duration were derived from the MCTQ (Jankowski 2017). In NCANDA, comparable but not identical measures were available. Rather than directly harmonizing MCTQ-derived chronotype, social jetlag, and sleep duration indices, we derived parallel indicators of sleep timing and alignment using the NCANDA Sleep Habits Questionnaire, a brief instrument administered across sites that assesses habitual bedtimes and wake times on school/work days and free days, as well as circadian preference. From these items, we derived measures of chronotype, relative social jetlag, and weighted average sleep duration, which together captured individual differences in sleep timing, misalignment, and quantity.

#### Self-Regulation and Cognition

Measures of executive function and impulsivity were harmonized across datasets where possible. In ABCD, performance-based executive function and crystallized abilities were assessed using NIH Toolbox age-corrected scores, including Flanker Inhibitory Control, List Sorting Working Memory, Pattern Comparison Processing Speed, Dimensional Change Card Sort (DCCS/Card Sort), Picture Vocabulary, and Oral Reading Recognition (Weintraub et al. 2014). Impulsivity traits were assessed via self-report using the UPPS-P subscales (sensation seeking, negative urgency, positive urgency, lack of perseverance, lack of planning) (Cyders et al. 2014). Behavioral decisionmaking was indexed using a delay discounting task (Odum 2011; Gelino et al. 2025), re-derived from the ABCD DDIS file as log10(k) using indifference-point proportions at the 6-hour, 1-day, 1-week, 1-month, 3-month, 1-year, and 5-year delays; ≥2 valid delays were required to compute an individual estimate, and Johnson & Bickel (JB) consistency screening was applied (Johnson and Bickel 2008), with values set to missing when JB criteria were violated.

In NCANDA, self-regulation was operationalized using a combination of self-reported impulsivity and executive functioning measures. Impulsivity traits were assessed using the UPPS-P subscales (sensation seeking, negative urgency, positive urgency, lack of perseverance, lack of planning) (Cyders et al. 2014). Executive functioning was indexed using informant-reported BRIEF subscales (inhibition, cognitive flexibility/shift, plan- ning/organization) (Huizinga et al. 2023) and performance-based measures capturing attention and working memory, including Stroop interference and error rates and Paced Auditory Serial Addition Test (PASAT) accuracy, error rates, and quit time (Diehr et al. 1998; Smolker et al. 2022).

#### Covariates

Age, sex, socioeconomic status, and study site were included as covariates. Socioeconomic status was indexed by household income or equivalent harmonized measures within each dataset.

Additional descriptions and psychometric properties of measures are provided in Supplementary Table 3.

### Analytic Approach

Analyses proceeded in two stages. In the discovery stage, we applied regularized canonical correlation analysis (rCCA) in ABCD to identify latent dimensions linking the sleep block (MCTQ chronotype, social jetlag, and sleep duration) and the cognition/self-regulation block (NIH Toolbox tasks, UPPS-P facets, delay discounting) (Hardoon et al. 2004; Witten et al. 2009; Nakua et al. 2024). Before analysis, covariates (age, sex, SES, site) were residualized from all predictors and variables were winsorized, imputed, and standardized. Although both ABCD and NCANDA include longitudinal followup, all canonical correlation analyses used a single baseline observation per participant, so each individual contributed only one row to the analyses. Because ABCD and NCANDA differ in developmental stage, we did not assume invariance of canonical weights across cohorts. CCA was conducted independently in each cohort, and cross-cohort comparisons focused on whether qualitatively similar latent dimensions emerged, rather than on the equivalence of coefficients.

Regularization parameters for the two variable blocks were selected by 5-fold cross-validation, choosing the penalty values that maximized the average held-out canonical correlation across folds and the same crossvalidation procedure was embedded within each permutation sample. For significance testing, we then performed permutation tests in which the sleep block was held fixed and the cognition/self-regulation block was permuted across participants; in each permuted dataset, we repeated the full cross-validation procedure to reestimate the penalty parameters before refitting rCCA, and the resulting canonical correlations were compared with the observed values to obtain permutation *p*-values. Structure loadings were bootstrapped with sign alignment to quantify the stability of each variable’s contribution.

To evaluate cross-cohort generalizability, we projected the ABCD-derived first canonical variate onto NCANDA using directly overlapping measures. Specifically, UPPS-P impulsivity subscales were aligned across cohorts, and three sleep indicators (chronotype, social jetlag, and sleep duration) were included in both datasets. Canonical weights estimated in ABCD were applied to standardized NCANDA data to compute projected sleep (U_1_) and self-regulation (V_1_) scores. Generalizability was quantified as the Pearson correlation between projected U_1_ and V_1_ scores in NCANDA.

### Statistical Software

All analyses were conducted in R (version 4.3.0) (R Core Team 2021). Canonical correlation analyses with regularization were implemented using the mixOmics package (Rohart et al. 2017). Data harmonization and preprocessing were performed using custom R scripts built on the data.table (Barrett et al. 2025) and dplyr (Wickham et al. 2023) frameworks. Multiple imputation was implemented with the mice package for ABCD (Buuren and Groothuis-Oudshoorn 2011), while median imputation was used for NCANDA, and resampling procedures (cross-validation, permutation, and bootstrapping) were carried out using rsample (Frick et al. 2025) and base R functions. Visualization of canonical scores and structure loadings was performed with ggplot2 (Wickham 2016).

### Study Approval and Ethics

This study involved secondary analysis of de-identified data from the ABCD and NCANDA consortia. Both studies obtained informed consent and assent from participants and their parents or guardians at the time of original data collection. Analyses of the harmonized datasets were reviewed by the University of California San Diego Institutional Review Board and determined to be exempt from further oversight due to the use of publicly available, de-identified data.

## Results

### ABCD Cohort

rCCA identified a modest but reliable multivariate association between sleep and cognition/self-regulation at baseline in the ABCD cohort. The first canonical function showed a full-sample canonical correlation of r_1_ = 0.21 (permutation *p* < 0.001), with variance extracted of 0.40 in the sleep block and 0.21 in the cognition/self- regulation block, yielding low but non-zero redundancy indices (YǁX = 0.01; XǁY = 0.02; Table 2). The second canonical function was substantially smaller (r_2_ = 0.07); although its permutation *p*-value was statistically significant, variance extracted and redundancy were near zero and no variables exceeded the ∣r∣ ≥.30 threshold, and therefore this function was not interpreted further.

**Figure 1.**
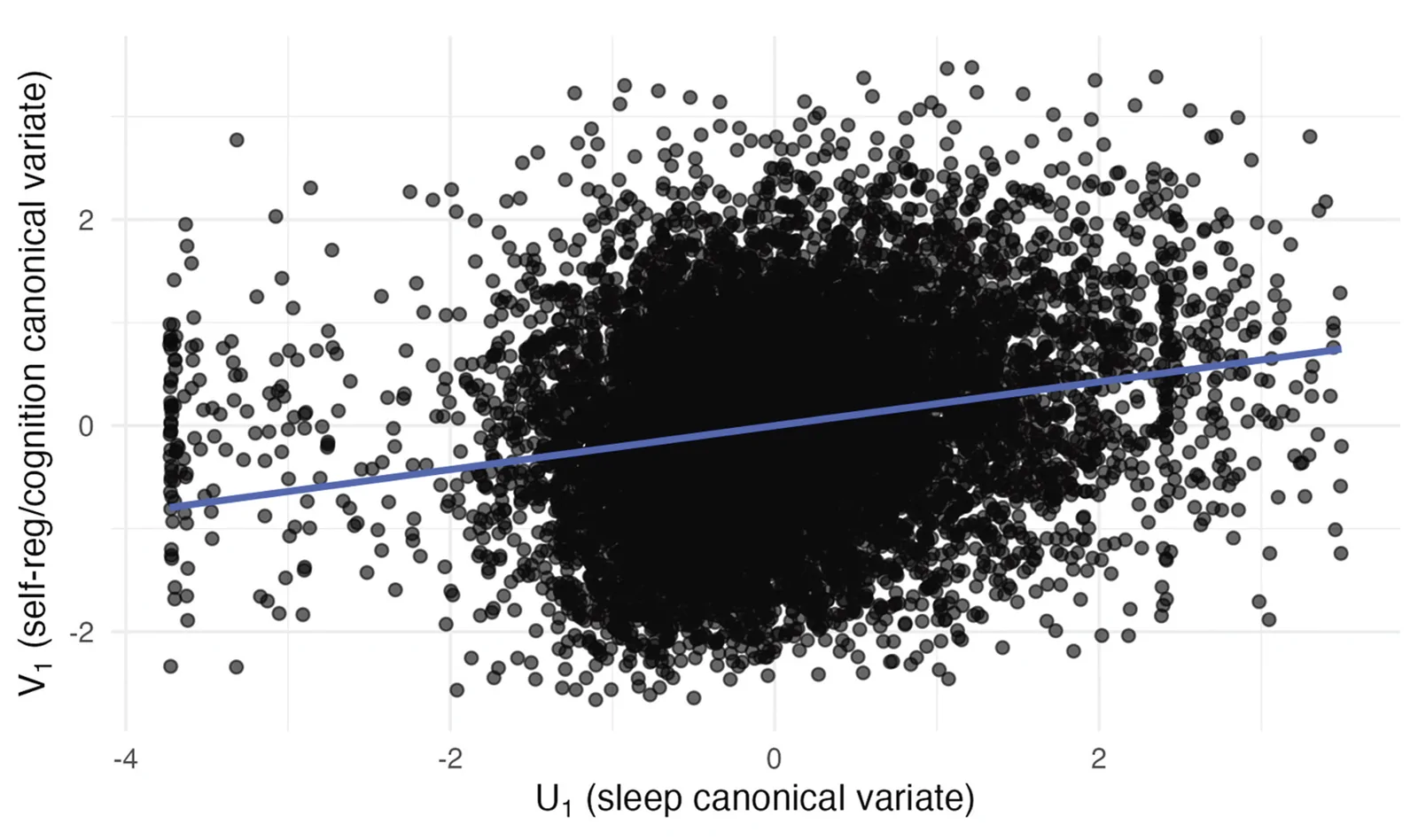
Scatterplot of canonical variates (sleep vs. cognition/self-regulation) for the first canonical function in ABCD, with fitted regression line. Scatterplot of canonical variates from the first canonical function in the ABCD cohort, illustrating the association between the sleep canonical variate (U1) and the self-regulation/cognition canonical variate (V1). Each point represents an individual participant. The fitted regression line is shown in blue. The observed canonical correlation (*r* = 0.21, permutation *p*-value < 0.001) indicates a modest but reliable multivariate association between sleep characteristics (dominated by chronotype and sleep duration) and self-regulatory functioning.

**Figure 2.**
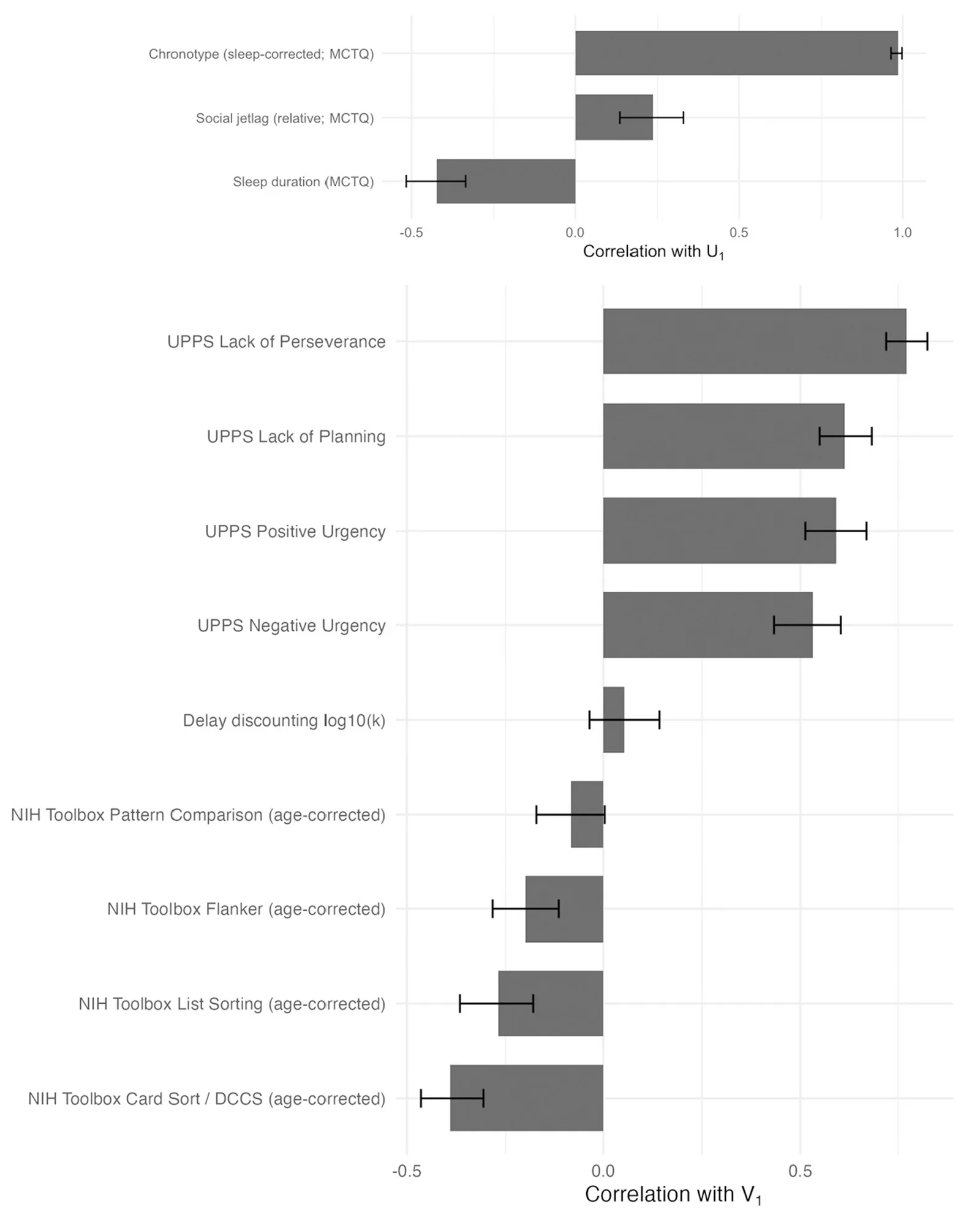
Structure loadings for the X (sleep variables) and Y (cognition/behavioral variables) blocks in ABCD. The first panel shows structure loadings for the X block (sleep variables; correlations with U1). The second panel shows structure loadings for the Y block (cognition and self-regulation variables; correlations with V1). Bars depict mean structure loadings and error bars indicate 95% bootstrap confidence intervals. Positive loadings indicate variables contributing in the same direction as the canonical variate; negative loadings indicate inverse contributions.

As shown in Figure 1, the association between the first pair of canonical variates (U1 and V1) is consistent but modest across individuals. Structure loadings for Function 1 (Figure 2) indicate that the sleep canonical variate (U1) is dominated by chronotype (sleep-corrected; MCTQ), with a strong positive loading, and shorter sleep duration, which loads negatively. Relative social jetlag did not exceed the ∣r∣ ≥.30 threshold. The corresponding cognition/self- regulation canonical variate (V1) loads most strongly on UPPS impulsivity facets, including lack of perseverance, lack of planning, positive urgency, and negative urgency, alongside a moderate negative loading for NIH Toolbox Card Sort/DCCS, reflecting poorer executive flexibility. Other cognitive measures, including List Sorting and delay discounting (log10[k]), contributed weakly and did not meet the loading threshold.

### NCANDA Cohort

Applying the same CCA framework to NCANDA at baseline yielded one significant canonical function (Table 3): r_1_ = 0.153, with subsequent functions not reaching statistical significance (r_2_ = 0.086, r_3_ = 0.074). Step-down Wilks’ lambda tests indicated significance for the full model including all three functions, but not for the reduced models excluding Function 1 (all→3: λ = 0.964, F(15, 2272.34) = 2.02, *p* = 0.011; 2→3: λ = 0.987, F(8, 1648.00) = 1.34, *p* = 0.220; 3→3: λ = 0.995, F (3, 825.00) = 1.51, *p* = 0.210). Accordingly, interpretation focuses on Function 1.

**Figure 3.**
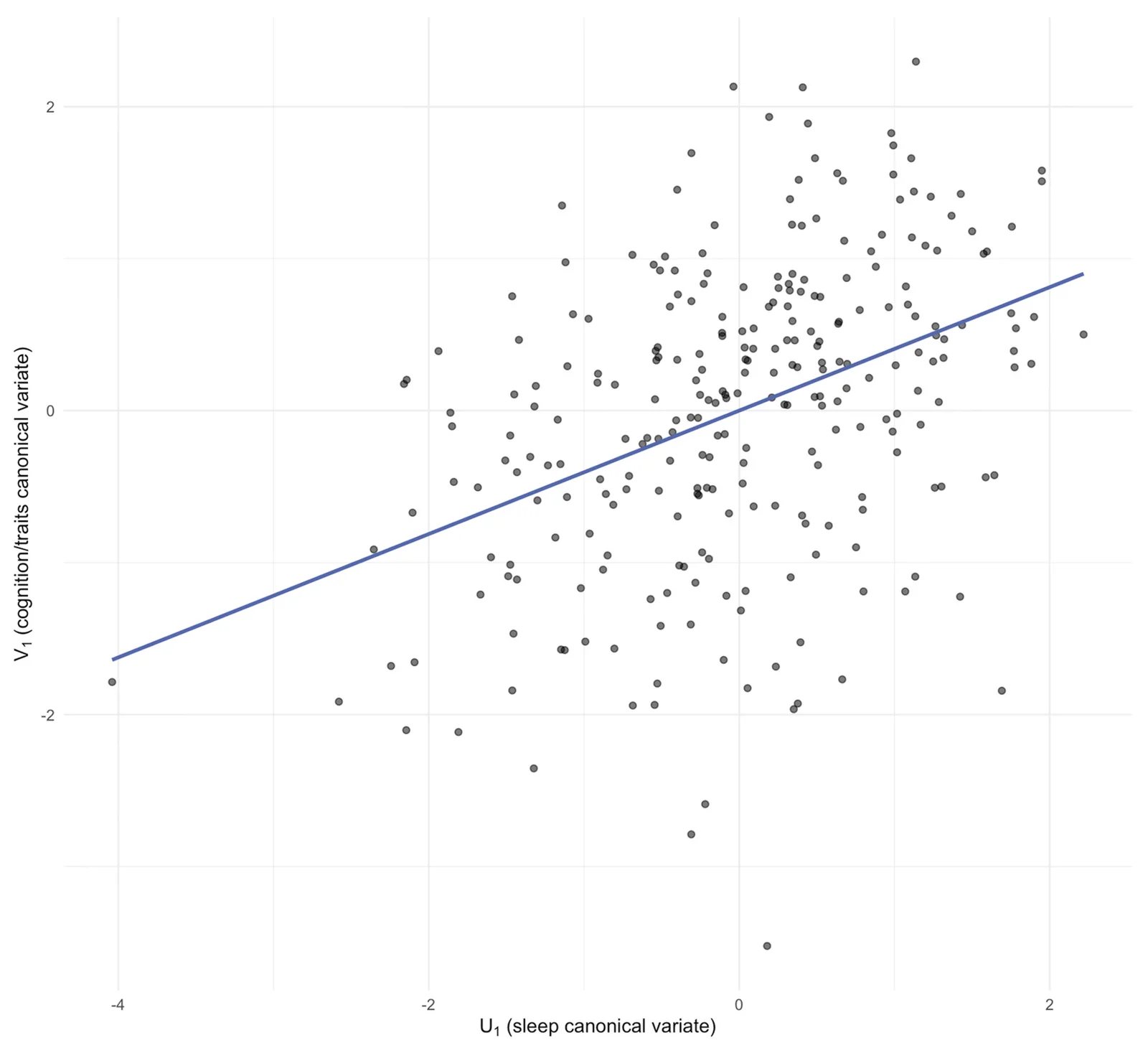
Scatterplot of canonical variates (sleep vs. cognition/self-regulation) for the first canonical function in NCANDA, with fitted regression line. Scatterplot of the first canonical function relating sleep variables to cognition/self-regulation in NCANDA (canonical correlation *r* = 0.153, *p*-value = 0.011), illustrating a modest but statistically significant association.

**Figure 4.**
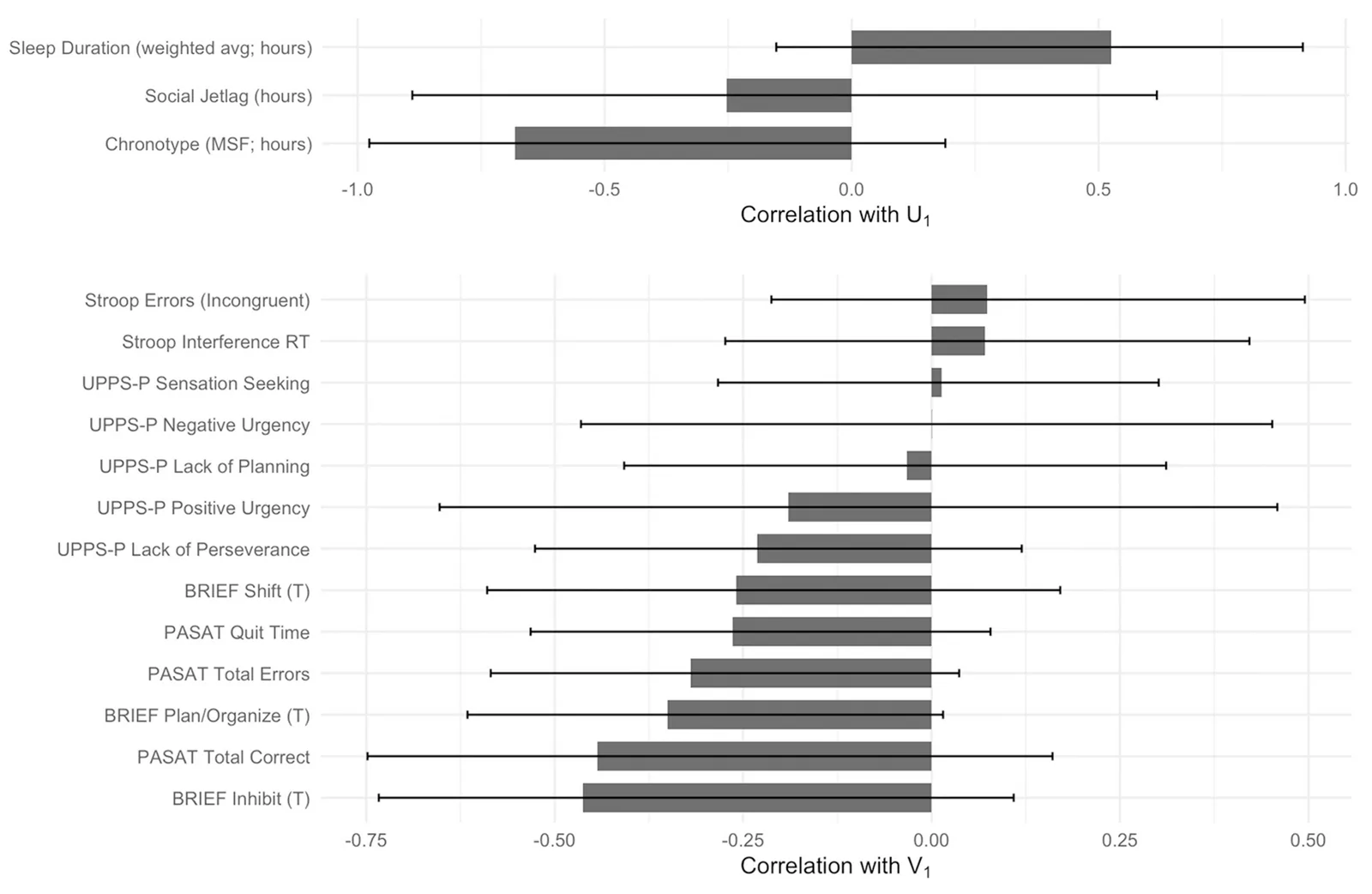
Structure loadings for the X (sleep variables) and Y (cognition/behavioral variables) blocks in NCANDA. First panel: correlations between sleep variables (Chronotype [MSF; hours], Social Jetlag [hours], and Sleep Duration [weighted average; hours]) and the sleep canonical variate U_1_. U_1_ is characterized by strong negative loadings for Chronotype and Social Jetlag, with minimal contribution from Sleep Duration, indicating a pattern driven primarily by later sleep timing and circadian misalignment. Second panel: correlations between cognition/behavioral variables and the self-regulation canonical variate V_1_. The largest magnitudes are observed for impulsivity-related measures, including UPPS-P Positive and Negative Urgency, along with moderate contributions from Lack of Perseverance and Lack of Planning. Additional contributions are observed for executive function measures, including BRIEF indices (Inhibit, Plan/Organize, Shift) and PASAT performance (Total Correct, Total Errors, Quit Time), whereas Stroop measures and Sensation Seeking show minimal contributions. Bars depict mean structure loadings and error bars indicate 95% bootstrap confidence intervals. Loading signs are arbitrary with respect to the direction of the canonical variates.

The canonical scatterplot for Function 1 is shown in Figure 3. Structure loadings are shown in Figure 4 and Table 3. In the X block (sleep), Chronotype (MSF; hours) loaded strongly and negatively on U_1_ (-0.970), accompanied by a moderate negative loading for Social Jetlag (hours) (-0.866) and minimal contribution from Sleep Duration (-0.039), indicating a sleep pattern primarily characterized by later timing and greater circadian misalignment rather than sleep duration.

In the Y block (cognition/behavior), the largest loadings on V_1_ reflected impulsivity dimensions, including strong negative loadings for UPPS-P Positive Urgency (-0.906) and Negative Urgency (-0.515), along with moderate contributions from Lack of Perseverance (-0.405) and Lack of Planning (-0.387). Sensation seeking did not contribute meaningfully. These results indicate that the NCANDA canonical variate is primarily driven by impulsivity-related processes rather than broader cognitive or achievement-related constructs.

Redundancy analysis indicated that the sleep canonical variates accounted for 0.7%, 0.2%, and 0.1% of variance in the cognition/behavior block for Functions 1–3, respectively (Redundancy YǁX = 0.007, 0.002, 0.001; total = 0.009). Conversely, the cognition/behavior variates accounted for 1.3%, 0.1%, and 0.2% of variance in the sleep block (Redundancy XǁY = 0.013, 0.001, 0.002; total = 0.016), consistent with a modest but interpretable crossdomain association driven primarily by the first canonical function.

### Projection Analysis

To evaluate cross-cohort generalizability, we projected the ABCD Function 1 canonical solution onto NCANDA by applying the ABCD-derived loadings to harmonized NCANDA indicators indexing overlapping sleep and self-regulation constructs. The projected sleep and self-regulation variates showed a modest but statistically significant association (*r* = 0.11, *p* < 0.001, *N* = 831), indicating partial generalization of the ABCD sleep – selfregulation coupling to an independent cohort.

Within NCANDA, the projected self-regulation variate (V1) was most strongly associated with social jetlag and chronotype, whereas associations with sleep duration were minimal. This pattern suggests that sleep – self-regulation coupling in NCANDA is driven more by circadian misalignment and timing than by total sleep duration.

To further evaluate structural similarity, we refit a ridge-regularized CCA model in NCANDA and compared canonical loadings across cohorts. Loadings on the sleep side showed moderate congruence with the ABCD solution (Tucker’s φ ≈ .61), whereas loadings on the self-regulation side showed poor congruence (φ ≈ -.27), indicating limited cross-cohort invariance of the self-regulation construct.

### Cross-Cohort Synthesis

Across ABCD and NCANDA, the leading sleep dimension consistently reflects later sleep timing, which – despite cohort-specific measurement differences – is reliably coupled with a self-regulation/impulsivity profile. In ABCD, later chronotype co-occurs with shorter sleep duration and shows a modest but robust association with self-regulation (r_1_ = 0.21; r_2_ trivial and not interpreted). In NCANDA, later sleep timing and greater sleep misalignment define a modest but significant first canonical function (r_1_ = 0.15), whereas higher- order functions are not statistically significant. Despite this weaker coupling in NCANDA, variance transfer (redundancy) remains small, indicating broad – but shallow – cross-block sharing rather than tight one-to- one correspondence between sleep and behavioral domains. The projection analysis further supports generalizability: when aligned impulsivity constructs are used across cohorts, the ABCD-derived variate reproduces a modest U – V association (*r* = 0.11), with chronotype and social jetlag carrying most of the sleep signal. Taken together, the evidence points to a consistent, cross-cohort link between delayed sleep timing and greater impulsivity, while also underscoring that the multivariate effects are modest and sensitive to measurement differences across studies.

## Discussion

In the present study, we examined multivariate associations between sleep and self-regulation in two large adolescent cohorts, ABCD and NCANDA, using regularized canonical correlation analysis with harmonized domains. Across both datasets, sleep characteristics – manifesting as later chronotype and shorter sleep duration in ABCD, and later sleep timing, greater social jetlag, and shorter sleep duration in NCANDA – were consistently associated with higher impulsivity and aspects of executive functioning. In ABCD, this pattern was most evident for UPPS-P urgency, lack of perseverance, and lack of planning, while delay discounting contributed minimally to the primary canonical function. Although delay discounting is often considered a reward-related construct, it captures a distinct aspect of behavior relative to affect-driven impulsivity. Experimental studies demonstrate that sleep deprivation increases impulsive action but does not reliably alter delay discounting, suggesting dissociable behavioral and neural mechanisms (Libedinsky et al. 2013; Demos et al. 2016). Consistent with this distinction, sleep disturbances are more robustly associated with impulsive behaviors in adolescents (Zhang et al. 2025), whereas delay discounting appears less sensitive to sleep-related variation and exhibits greater measurement variability (O’Connor et al. 2018).

Our findings are broadly consistent with dualsystems and sleep – emotion regulation models, in which sleep disruption preferentially affects affect- driven impulsivity via altered prefrontal – limbic dynamics (Yoo et al. 2007; Casey et al. 2008; Steinberg 2008; Somerville and Casey 2010; Goldstein and Walker 2014). Future work could directly test these mechanisms by integrating neuroimaging markers of frontostriatal and frontoamygdala circuitry and by examining whether improving sleep timing or reducing circadian misalignment attenuates urgency-related impulsivity.

In NCANDA, the strongest associations were observed for chronotype and sleep misalignment in the sleep block, coupled with impulsivity and executive functioning correlates, including UPPS-P urgency facets, BRIEF indices, and PASAT performance, reflecting inhibitory control and working memory processes. These NCANDA findings are consistent with prior experimental and observational work demonstrating that insufficient or misaligned sleep impairs inhibitory control, working memory, and cognitive flexibility (Anderson et al. 2009; Beattie et al. 2015), and with evidence that circadian timing variables such as chronotype and social jetlag may be more closely linked to impulsivity and self-regulation than sleep duration alone (Owens et al. 2016; Logan et al. 2018; Hasler et al. 2022; Panjeh et al. 2025).

These results build directly on prior research. Our recent analysis of ABCD data demonstrated that chronotype, especially in the context of stress exposure, relates to higher impulsivity on UPPS-P urgency dimensions (McCarthy et al. 2023). The present study extends those findings by showing that these relations persist within a multivariate canonical structure spanning multiple facets of sleep and self-regulation. Likewise, prior evidence that social jetlag is associated with poorer crystallized cognition and academic performance in ABCD (Li et al. 2024) is conceptually consistent with the current observation that later sleep timing aligns with executive dysfunction. Taken together, the emerging picture is that circadian timing and insufficient sleep during adolescence are linked to broader self-regulatory difficulties.

Notably, in ABCD only the first canonical function was practically meaningful: Function 1 showed a modest but reliable correlation and non-trivial redundancy, whereas Function 2 had a small correlation, near-zero redundancy, and no variables exceeding the ∣r∣ ≥.30 threshold. In NCANDA, only the first canonical function was statistically and practically meaningful, with the dominant sleep dimension marked by later chronotype, greater social jetlag, and shorter sleep duration, aligning with poorer self-regulation across impulsivity and executive function measures. This pattern is consistent with the structure of canonical correlation analysis, in which the first function captures the dominant shared variance between domains, whereas subsequent functions reflect increasingly residual and orthogonal variance that is often weaker and less stable. In the present context, sleep and self-regulation appear to share a single primary axis of association – characterized by later sleep timing and greater circadian misalignment aligning with impulsivity-related processes – rather than multiple independent modes of coupling.

Additional canonical functions likely capture noise, measurement-specific variance, or cohort-specific structure that does not generalize across samples, which is consistent with their low redundancy and lack of stable loadings. Effect sizes for redundancy were modest in both cohorts, indicating that while the coupling is consistent, it explains a limited portion of cross-block variance.

We further evaluated cross-cohort generalizability by projecting the ABCD Function-1 canonical loadings into NCANDA using overlapping sleep and impulsivity measures. Within NCANDA, the projected selfregulation variate was most strongly related to social jetlag and later chronotype, whereas associations with sleep duration were minimal, suggesting that circadian misalignment rather than total sleep time primarily drives the transferred signal. When the CCA model was refit in NCANDA, sleep-side loadings showed moderate congruence with the ABCD solution, whereas selfregulation loadings showed poor congruence, consistent with limited cross-cohort invariance of the selfregulation dimension. Together, these findings suggest that aspects of the sleep structure – particularly timing and misalignment – generalize across cohorts, but that differences in developmental stage, measurement batteries, and construct organization on the self-regulation side likely attenuate the strength of cross-cohort projection.

Several limitations should be acknowledged. Both cohorts rely largely on self-report for sleep, which may introduce recall bias relative to actigraphy or polysomnography (Meltzer et al. 2012). Harmonization was necessarily at the domain level, and some ABCD measures (e.g. NIH Toolbox tasks and MCTQ timing) did not have direct NCANDA counterparts, necessitating imperfect alignment of indicators across cohorts; remaining differences in measurement and construct representation likely contribute to attenuation of crosscohort projection effects.

Analyses were cross-sectional at baseline, limiting causal inference; self-regulatory problems may exacerbate sleep difficulties, and unmeasured third variables (e.g. ADHD, depression, medication use, psychosocial stress) could drive associations in both domains. Finally, large samples increase power to detect modest effects, so practical significance should be weighed using redundancy and external validity alongside statistical significance.

Future directions include longitudinal modeling to clarify temporal precedence and downstream outcomes (e.g. substance use, mood, and academic performance), incorporation of objective sleep measures (e.g. acti- graph) to improve precision (Bei et al. 2016), and tests of moderation by sex, socioeconomic status, and cultural context. Mechanistic work – including neuroimaging – may help specify pathways linking eveningness and reduced sleep duration to executive control deficits and reward sensitivity (Holm et al. 2009).

These findings have potential policy and intervention implications. The observed association between circadian timing and self-regulation supports ongoing efforts to align adolescent schedules with biological sleep patterns, including later school start times, which have been shown to improve sleep duration and daytime functioning (Wahlstrom 2014; Wheaton et al. 2016; Ziporyn et al. 2022; Mousavi and Troxel 2023). At the individual level, interventions targeting sleep timing and circadian alignment may be particularly relevant. These include behavioral strategies such as consistent sleep – wake scheduling, reduction of evening light exposure (e.g. screen use), and appropriately timed morning light exposure to advance circadian phase (Chellappa 2020; Brown et al. 2022; Wallace et al. 2025). More structured approaches, including chronotherapy or light-based interventions, may also hold promise for improving self-regulatory functioning by reducing circadian misalignment (Emens and Burgess 2015). Although the observed effect sizes are modest, these interventions are scalable and low-risk, suggesting potential population-level benefits.

Taken together, the present findings demonstrate reproducible, multivariate associations between sleep timing/alignment and self-regulation across two large cohorts. In ABCD, later sleep timing and shorter sleep duration were linked to impulsivity-laden self-regulation; in NCANDA, a modest pattern emerged linking later sleep timing and greater social jetlag with UPPS-P impul- sivity. Projection results, based on directly overlapping UPPS-P impulsivity measures and parallel sleep timing/ duration indicators, point in the same direction. These findings extend prior work (McCarthy et al. 2023; Li et al. 2024) and strengthen the case that sleep health is relevant to adolescent self-regulation and a promising target for prevention and intervention.

## Supplementary Material

Supp 1

Supplemental data for this article can be accessed online at https://doi.org/10.1080/07420528.2026.2685032

## Figures and Tables

**Table 1.** Baseline sociodemographic characteristics of the ABCD and NCANDA analytic cohorts.

Characteristic	ABCD	NCANDA	Δ (ABCD – NCANDA)	Test (df)	*^p^*	Effect size
*Sex* (n)	11,143	831	—	X^2^ = 3.59 (1)	0.058	Cramér’s V = 0.017
Female	5,293 (47.5%)	423 (50.9%)	−3.4 pp	—	—	—
Male	5,850 (52.5%)	408 (49.1%)	+3.4 pp	—	—	—
SES (min – max harmonized)	mean 0.764 (SD 0.248), *n* = 10,168	mean 0.702 (SD 0.221), *n* = 831	+0.062	Welch *t* = 7.68 (≈1009.1)	3.69 × 10^−1 4^	Cohen’s d = 0.263
*Race/Ethnicity* (n)	11,143	831	—	X^2^ = 796.76 (7)	9.26 × 10^−1 6 8^	Cramér’s V = 0.258
Asian	212 (1.9%)	61 (7.3%)	−5.4 pp	—	—	—
Black/African American	1,623 (14.6%)	99 (11.9%)	+2.7 pp	—	—	—
Hispanic/Latino	2,251 (20.2%)	97 (11.7%)	+8.5 pp	—	—	—
Multiracial/Other	1,128 (10.1%)	0 (0.0%)	+10.1 pp	—	—	—
Other	0 (0.0%)	36 (4.3%)	−4.3 pp	—	—	—
White	5927 (532%)	532 (640%)	−108 pp	—	—	—

This table summarizes demographic and socioeconomic characteristics of participants included in the baseline analytic cohorts of the Adolescent Brain Cognitive Development (ABCD) and National Consortium on Alcohol and Neurodevelopment in Adolescence (NCANDA) cohorts. Variables include sex, race/ ethnicity, and socioeconomic status (SES; min – max harmonized). Percentages represent within-cohort proportions; differences (Δ) are in percentage points for categorical variables and raw mean differences for continuous variables. Group differences were evaluated using chi-square tests for categorical variables and Welch’s t-tests for continuous measures, with effect sizes reported as Cramér’s V and Cohen’s d, respectively.

**Table 2.** ABCD CCA results.

Measure	Function 1	Function 2
Canonical correlation	0.21	0.07
Permutation p-value	<0.001	<0.001
Variance extracted (X)	0.40	0.42
Variance extracted (Y)	0.21	0.14
Redundancy Y‖X	0.01	0.00
Redundancy X‖Y	0.02	0.00
Key structure loadings (∣r∣ ≥0.30)		
Chronotype (sleep-corrected; MCTQ)	0.99	—
UPPS Lack of Perseverance	0.78	—
UPPS Lack of Planning	0.61	—
UPPS Positive Urgency	0.59	—
UPPS Negative Urgency	0.54	—
NIH Toolbox Card Sort / DCCS (age-corrected)	−0.40	—
Sleep duration (MCTQ)	−0.42	—

Canonical correlations, permutation-based significance tests, variance extracted, redundancy indices, and structure loadings from canonical correlation analysis relating sleep characteristics and self-regulation/cognition in the ABCD Study. Function 1 shows a modest but reliable multivariate association (*r* = 0.21), primarily reflecting later chronotype and shorter sleep duration in relation to higher impulsivity and poorer executive flexibility. Function 2 shows a very small canonical correlation (*r* = 0.07) with negligible redundancy; despite statistical significance due to large sample size, this function was not interpreted. Structure loadings represent variable – canonical variate correlations, sign-aligned to the first sleep (U1) and self-regulation/cognition (V1) canonical variates.

**Table 3.** NCANDA CCA results.

Measure	Function 1	Function 2	Function 3
Canonical correlation	0.153	0.086	0.074
Wilks’ λ	0.964	0.987	0.995
Approx. F	2.02	1.34	1.51
df1	15	8	3
df2	2272.34	1648.00	825.00
p-value	0.011	0.220	0.210
Variance extracted (X)	0.508	0.253	0.239
Variance extracted (Y)	0.050	0.094	0.050
Redundancy Y‖X	0.007	0.002	0.001
Redundancy X‖Y	0.013	0.001	0.002
Key structure loadings (*r* ≥ 0.30)			
Chronotype (MSF; hours)	−0.970	—	—
Social Jetlag (hours)	−0.866	—	—
Sleep Duration (weighted avg; hours)	—	—	—
UPPS-P Positive Urgency	−0.906	—	—
UPPS-P Negative Urgency	−0.515	—	—
UPPS-P Lack of Planning	−0.387	—	—
UPPS-P Lack of Perseverance	−0.405	—	—
BRIEF Plan/Organize (T)	−0.34	—	—
BRIEF Inhibit (T)	−0.45	—	—
BRIEF Shift (T)	−0.30	—	—
PASAT Total Correct	−0.30	—	—
PASAT Total Errors	−0.32	—	—
PASAT Quit Time	−0.31	—	—

Canonical correlations, Wilks’ lambda tests, redundancy indices, and structure loadings (absolute value ≥ 0.30) for the first three canonical functions in the NCANDA sample. Only the first canonical function was statistically significant and is interpreted. Sleep variables included chronotype (midsleep on free days), social jetlag, and weighted average sleep duration (all expressed in hours). Loadings reflect correlations of observed variables with the corresponding canonical variates (sleep → U, cognition/ self-regulation → V). Loading signs are arbitrary with respect to the orientation of the canonical variates.

## Data Availability

Data used in this study were obtained from the Adolescent Brain Cognitive Development (ABCD) Study (https://abcdstudy.org), a longitudinal study funded by the National Institutes of Health under awards U01DA041022, U01DA041028, U01DA041048, U01DA041089, U01DA041106, U01DA041117, U01DA041120, U01DA041134, U01DA041148, U01DA041156, U01DA041174, U24DA041123, and U24DA041147. Data from the National Consortium on Alcohol and Neurodevelopment in Adolescence (NCANDA) were accessed through the NIAAA Data Archive (collection 4513; https://nda.nih.gov/edit_collection.html?id=4513). Both datasets are available to qualified investigators through formal data access requests and are subject to the respective data use agreements.
