# Supplementary material for: Multivariate associations between sleep patterns and self-regulation in adolescence: Canonical correlation analysis across ABCD and NCANDA cohorts
Source: Chronobiol Int. Author manuscript; Available in PMC 2026 Aug 11. (PMC13455002; doi:10.1080/07420528.2026.2685032)
Supplement: Supplementary file 1 [file NIHMS2197356-supplement-Supp_1.docx]

**Supplementary Material**

**Supplementary Table 1: ABCD vs NCANDA Measures Mapping**

| **Domain** | **Construct** | **ABCD variable(s)** | **ABCD instrument/source** | **NCANDA variable(s)** | **NCANDA instrument/source** | **Harmonization notes** |
| --- | --- | --- | --- | --- | --- | --- |
| Sleep timing | Chronotype (sleep-corrected) | chronotype_msf | MCTQ (youth) | chronotype_msf_min | Sleep Behavior Questionnaire (derived) | Both cohorts capture individual differences in sleep timing/chronotype; measures are conceptually aligned but derived from different instruments. |
| Sleep timing / alignment | Relative social jetlag (sleep-corrected) | sjl_sc | Derived from MCTQ | sjl_min | Sleep Behavior Questionnaire (derived) | Both cohorts index the magnitude of weekday–weekend sleep timing misalignment; definitions differ slightly by instrument. |
| Sleep duration | Average sleep duration | sleep_duration_avg | Derived (weekday/weekend reports) | sleep_duration_wt_min | Sleep Behavior Questionnaire (derived) | Both cohorts use weighted weekday/weekend averages to index habitual sleep duration. |
| Executive function / attention (performance) | Inhibitory control & attention | nihtbx_flanker_agecorrected | NIH Toolbox | stroop_interference_rt, stroop_errors_incong, pasat_total_correct | Stroop; PASAT | Approximate construct-level alignment on attention and inhibition using performance-based tasks. |
| Executive function / working memory | Working memory | nihtbx_list_agecorrected | NIH Toolbox | pasat_total_correct, pasat_total_errors, pasat_quit_time | PASAT | Conceptual mapping of working memory demands across tasks. |
| Cognitive flexibility / set-shifting | Set-shifting | nihtbx_cardsort_agecorrected | NIH Toolbox | brief_shift_t, brief_bri_t, brief_gec_t | BRIEF (ratings) | ABCD performance-based vs NCANDA ratings; convergent executive constructs. |
| Impulsivity (UPPS-P) | Negative Urgency | upps_negative_urgency | UPPS-P (Cyders et al., 2014) | upps_negative_urgency | UPPS-P | Directly aligned. |
| Impulsivity (UPPS-P) | Positive Urgency | upps_positive_urgency | UPPS-P | upps_positive_urgency | UPPS-P | Directly aligned. |
| Impulsivity (UPPS-P) | Lack of Perseverance | upps_lack_of_perseverance | UPPS-P | upps_lack_of_perseverance | UPPS-P | Directly aligned. |
| Impulsivity (UPPS-P) | Lack of Planning | upps_lack_of_planning | UPPS-P | upps_lack_of_planning | UPPS-P | Directly aligned. |
| Impulsivity (UPPS-P) | Sensation Seeking | upps_sensation_seeking | UPPS-P | upps_sensation_seeking | UPPS-P | Directly aligned. |
| Covariates | Demographics / SES / site | age, sex, SES, site | Cohort demographics | visit_age, sex, SES, site | Cohort demographics | Age, sex, SES (income-based/harmonized), and site included as covariates. |

In ABCD, sleep timing, relative social jetlag (sleep-corrected) (Jankowski, 2017), and average sleep duration were derived from the MCTQ. In NCANDA, parallel indices of chronotype, social jetlag, and sleep duration were derived from the Sleep Habits Questionnaire. Executive functioning in ABCD was assessed using NIH Toolbox tasks, while NCANDA included BRIEF subscales and performance-based measures such as Stroop and PASAT. Constructs were aligned at the construct level, with direct correspondence for UPPS-P measures and parallel definitions for sleep indicators. Covariates included age, sex, SES, and study site.

**Supplementary Table 2: Missingness and Multicollinearity Diagnostics in ABCD and NCANDA Cohorts**

| **Cohort** | **Variable** | **% Missing** | **Key Missingness Associations** | **VIF** |
| --- | --- | --- | --- | --- |
| **ABCD** | nihtbx_cardsort_agecorrected | 64.47% | Age, site, SES (p < .01) | — |
| ABCD | nihtbx_list_agecorrected | 27.54% | Age, site (p < .001) | — |
| ABCD | delay_discounting_logk | 17.36% | Age, SES (p < .001), sex (p < .01) | — |
| ABCD | ph_y_mctq_chrono | 16.64% | Sex (p < .05) | — |
| ABCD | nihtbx_pattern_agecorrected | 16.58% | Age, SES (p < .001) | — |
| ABCD | nihtbx_flanker_agecorrected | 16.40% | Age, SES (p < .01) | — |
| ABCD | ph_y_mctq__sleep_dur | 8.79% | Age (p < .05) | — |
| ABCD | ph_y_mctq__socjl_rel | 8.75% | None | — |
| ABCD | UPPS variables | 8.75% | None | — |
| ***ABCD*** | *All variables* | *—* | *Consistent with MAR* | ***Mean = 1.33; Max = 1.53*** |
| **NCANDA** | chronotype_msf_min | 65.82% | SES (p < 1e-7) | — |
| NCANDA | sjl_min | 65.82% | SES (p < 1e-7) | — |
| NCANDA | sleep_duration_wt_min | 65.82% | SES (p < 1e-7) | — |
| NCANDA | stroop_interference_rt | 3.49% | Sex (p < .01) | — |
| NCANDA | brief variables | 1.20% | None | — |
| NCANDA | UPPS variables | 0.24% | None | — |
| NCANDA | PASAT variables | 0.24% | Weak SES/age (ns) | — |
| ***NCANDA*** | *All variables* | *—* | *Consistent with MAR* | ***Mean = 2.36; Max = 6.38*** |

Variable-level missingness and multicollinearity diagnostics for the ABCD and NCANDA cohorts. Variable names are defined in Supplementary Table 1 above. Missingness percentages reflect the proportion of observations with missing values. In ABCD, missingness was variable-specific and most pronounced in certain NIH Toolbox tasks, whereas sleep and impulsivity measures exhibited moderate missingness. Missingness was systematically associated with observed covariates, particularly age and study site, consistent with a missing-at-random framework and supporting the use of multivariate imputation by chained equations (MICE). In NCANDA, missingness was concentrated in sleep variables (~66%), reflecting the subset of participants with available sleep data, whereas self-regulation measures exhibited minimal missingness. Missingness in NCANDA sleep variables was associated with socioeconomic status but not with age or sex, also consistent with missing-at-random assumptions. Multicollinearity was assessed using variance inflation factors (VIF), which were within acceptable ranges across both datasets (Craney and Surles, 2002).

**Supplementary Table 3. Description and Psychometric Properties of Study Measures**

| **Domain** | **Measure** | **Type** | **Description** | **Psychometric Properties** |
| --- | --- | --- | --- | --- |
| Sleep | MCTQ | Self-report | Assesses sleep timing, chronotype, and social jetlag via weekday/weekend sleep schedules | Self-report measure of sleep timing and chronotype with moderate longitudinal test–retest reliability for chronotype indices (r ≈ 0.51–0.58) and lower test–retest reliability for weekday sleep timing (r ≈ 0.29–0.40) (Jankowski, 2017; Roenneberg et al., 2004; Santisteban et al., 2018; Wang et al., 2023) |
| Sleep | Sleep Habits Questionnaire | Self-report | Assesses habitual sleep timing and duration across weekdays and weekends | Questionnaire-based measure of habitual sleep behaviors and sleep problems with adequate internal consistency (α ≈ 0.68–0.78 for total score; subscales ≈ 0.36–0.93) and acceptable test–retest reliability (r ≈ 0.62–0.79), supporting its validity as a screening tool for sleep disturbances in children (Brown et al., 2015; Owens et al., 2000) |
| Impulsivity | UPPS-P | Self-report | Five-factor impulsivity model (urgency, perseverance, planning, sensation seeking) | Self-report measure of multidimensional impulsivity (urgency, perseverance, planning, sensation seeking) with good internal consistency (α ≈ 0.74–0.88 across subscales), supporting reliable assessment of impulsivity traits (Cyders et al., 2014) |
| Executive function | NIH Toolbox | Performance-based | Computerized tasks assessing attention, working memory, cognitive flexibility | Performance-based cognitive battery with good test–retest reliability (ICC ≈ 0.66–0.87 across tasks and composite scores) (Karr et al., 2024; Weintraub et al., 2014) |
| Executive function | BRIEF | Informant-report | Behavioral ratings of executive functioning in real-world settings | Informant-report measure of executive functioning in everyday settings with high internal consistency (α ≈ 0.80–0.98) and strong test–retest reliability (ICC ≈ 0.75–0.90), indicating excellent reliability (Gioia et al., 2000; Jacobson et al., 2020) |
| Executive function | Stroop | Performance-based | Measures inhibitory control and interference processing | Well-validated experimental measure with moderate to strong reliability (r ≈ 0.60–0.80 for interference scores) (Emek-Savas et al., 2019; Scarpina and Tagini, 2017) |
| Executive function | PASAT | Performance-based | Measures working memory and processing speed under cognitive load | Well-validated measure of working memory and processing speed with strong test–retest reliability (r ≈ 0.73–0.90) across short and longer intervals, though performance is susceptible to practice effects (Diehr et al., 1998; Tombaugh, 2006) |
| Decision-making | Delay discounting | Behavioral task | Assesses preference for immediate vs delayed rewards (log k) | Behavioral measure of impulsive decision-making with moderate to high test–retest reliability in adolescence (r ≈ 0.67–0.76), supporting stability of individual differences (Anokhin et al., 2015; Johnson and Bickel, 2008; Odum, 2011) |

This table summarizes the primary sleep and self-regulation measures used in the present analyses, including instrument type, construct assessed, and key psychometric properties. Measures include self-report (e.g., Munich Chronotype Questionnaire [MCTQ], Sleep Habits Questionnaire, UPPS-P), informant-report (e.g., Behavior Rating Inventory of Executive Function [BRIEF]), and performance-based tasks (e.g., NIH Toolbox cognition battery, Stroop, Paced Auditory Serial Addition Test [PASAT], delay discounting). Across instruments, measures demonstrate acceptable to strong reliability and validity in adolescent and young adult populations, supporting their use in multivariate analyses of sleep and self-regulation. Psychometric properties are drawn from prior validation studies and are provided for contextualization rather than representing estimates from the present samples.
